# Icariside I specifically facilitates ATP or nigericin-induced NLRP3 inflammasome activation and causes idiosyncratic hepatotoxicity

**DOI:** 10.1186/s12964-020-00647-1

**Published:** 2021-02-11

**Authors:** Yuan Gao, Guang Xu, Li Ma, Wei Shi, Zhilei Wang, Xiaoyan Zhan, Nan Qin, Tingting He, Yuming Guo, Ming Niu, Jiabo Wang, Zhaofang Bai, Xiaohe Xiao

**Affiliations:** 1 School of Traditional Chinese Medicine, Capital Medical University, Beijing, 100069 China; 2 China Military Institute of Chinese Materia, the Fifth Medical Centre, Chinese PLA General Hospital, No. 100 Xisihuan, Beijing, 100039 China; 3 Integrative Medical Center, the Fifth Medical Centre, Chinese PLA General Hospital, Beijing, 100039 China

**Keywords:** *Epimedii folium*, Icariside I, Idiosyncratic drug-induced liver injury, NLRP3 inflammasome

## Abstract

**Background:**

Epimedii Folium (EF) is commonly used for treating bone fractures and joint diseases, but the potential hepatotoxicity of EF limits its clinical application. Our previous study confirms that EF could lead to idiosyncratic drug-induced liver injury (IDILI) and hepatocyte apoptosis, but the mechanism remains unknown. Studies have shown that NLRP3 inflammasome plays an important role in the development of various inflammatory diseases such as IDILI. Specific stimulus-induced NLRP3 inflammasome activation may has been a key strategy for lead to liver injury. Therefore, main compounds derived from EF were chosen to test whether the ingredients in EF could activate the NLRP3 inflammasome and to induce IDILI.

**Methods:**

Bone-marrow-derived macrophages (BMDMs) were treated with Icariside I, and then stimulated with inflammasome stimuli and assayed for the production of caspase-1 and interleukin 1β (IL-1β) and the release of lactate dehydrogenase (LDH). Determination of intracellular potassium, ASC oligomerization as well as reactive oxygen species (ROS) production were used to evaluate the stimulative mechanism of Icariside I on inflammasome activation. Mouse models of NLRP3 diseases were used to test whether Icariside I has hepatocyte apoptosis effects and promoted NLRP3 inflammasome activation in vivo.

**Results:**

Icariside I specifically enhances NLRP3 inflammasome activation triggered by ATP or nigericin but not SiO2, poly(I:C) or cytosolic LPS. Additionally, Icariside I does not alter the activation of NLRC4 and AIM2 inflammasomes. Mechanically, Icariside I alone does not induce mitochondrial reactive oxygen species (mtROS), which is one of the critical upstream events of NLRP3 inflammasome activation; however, Icariside I increases mtROS production induced by ATP or nigericin but not SiO2. Importantly, Icariside I leads to liver injury and NLRP3 inflammasome activation in an LPS-mediated susceptibility mouse model of IDILI, but the effect of Icariside I is absent in the LPS-mediated mouse model pretreated with MCC950, which is used to mimic knockdown of NLRP3 inflammasome activation.

**Conclusions:**

Our study reveals that Icariside I specifically facilitates ATP or nigericin-induced NLRP3 inflammasome activation and causes idiosyncratic hepatotoxicity. The findings suggest that Icariside I or EF should be avoided in patients with diseases related to ATP or nigericin-induced NLRP3 inflammasome activation, which may be risk factors for IDILI.

Video abstract.

**Supplementary Information:**

The online version contains supplementary material available at 10.1186/s12964-020-00647-1.

## Background

Idiosyncratic drug-induced liver injury (IDILI) is an adverse drug reaction that occurs in a minority of patients exposed to drugs, and its occurrence depends on individual susceptibility [1–3]. Therefore, IDILI is difficult to effectively assess by conventional preclinical drug safety evaluation models [4]. Recently, evidence indicating that IDILI mediated by immune responses has come to light, and many mechanisms have been proposed [5, 6]. Increasing evidence illustrate that a mild inflammatory microenvironment caused by inflammatory mediators, such as lipopolysaccharide (LPS) and TNF-α, could enhance susceptibility to the hepatotoxicity of several drugs with the ability to induce IDILI, such as trovafloxacin or chlorpromazine, and thereby result in idiosyncratic responses [7–9]. In addition, studies have shown that treating PD-1 knockout mice with amodiaquine and anti-CTLA4 leads to liver injury similar to IDILI [10]. With the widespread use of herbal and dietary supplements (HDS) worldwide, several researches have indicated that traditional Chinese medicine (TCM) herbal and dietary supplements are the leading causes of drug-induced liver injury (DILI), not only in Asian countries but also in western countries [11]. In particular, liver injury induced by TCMs, such as *Epimedii Folium, Psoraleae Fructus, Polygonum multiflorum* and *Dictamni Cortex*, have been reported frequently in mainland China [12–15]. Although accumulating evidence has demonstrated that some TCMs could induce IDILI, the mechanism is still unclear.The NLRP3 (the nucleotide binding domain and leucine-rich repeat (NLR) pyrin domain containing 3) inflammasome is a multiple protein complex consisting of NLRP3, apoptosis-associated speck-like protein containing CARD (ASC) and cysteinyl aspartate-specific proteinase-1 (caspase-1) that can be activated by pathogen-associated molecular patterns (PAMPs) and danger-associated molecular patterns (DAMPs), leading to the cleavage of pro-caspase-1. Once activated, caspase-1 promotes pyroptosis and the cleavage of pro-IL-1β and pro-IL-18 to produce mature and functional IL-1β and IL-18 [16, 17]. The NLRP3 inflammasome could drive a variety of inflammatory reactions, so persistent and aberrant NLRP3 inflammasome activation contributes to many chronic and degenerative diseases, including type 2 diabetes, Gout, atherosclerosis, Alzheimer’s disease, osteoarthritis, obesity, lupus, macular degeneration, and liver disease [18–20]. Previous studies have also shown that some chemical drugs with the ability to induce IDILI also cause NLRP3 inflammasome activation in vitro by inducing the release of DAMPs from damaged or dead cells, suggesting that NLRP3 inflammasome activation may be a critical mechanism of some drug-mediated idiosyncratic liver injury [19]. However, whether TCMs with the ability to induce IDILI may also induce liver injury by activating NLRP3 inflammasomes remains to be studied.

*Epimedii Folium (EF)* is a famous herbal medicine that contains several medically active constituents, including flavonoids and phytosteroids, which are commonly used in China, Japan, and Korea [21]. Nevertheless, with numerous reports about liver injury related to EF, more attention has been paid to the clinical safety of EF. Thus, it is urgent to elucidate the characteristics and mechanisms of EF-induced liver injury [22, 23]. Our previous studies confirmed that EF could induce liver injury in an LPS-mediated susceptible mouse model of IDILI. In this model, liver injury was accompanied by elevated serum levels of IL-1β [14], so we speculate that components derived from EF may induce liver injury by promoting the formation of NLRP3 inflammasome.

In this study, our data indicate that Icariside I, which is one of the major metabolic constituents of EF, can specifically increase ATP and nigericin-induced NLRP3 inflammasome activation to inflame the liver and cause idiosyncratic hepatotoxicity.

## Methods

### Mice

Female 6–8-week-old C57BL/6 mice were purchased from SPF Biotechnology Co., Ltd. (Beijing, China). All animals were maintained under 12-h light/dark conditions at 22 °C–24 °C with unrestricted access to food and water for the duration of the experiment. All animal protocols in this study were performed according to the guidelines for care and use of laboratory animals and approved by the animal ethics committee of the Fifth Medical Centre, Chinese PLA (People’s Liberation Army) General Hospital (animal ethics committee approval No. IACUC-2017-003).

### Reagents and antibodies

Adenosine triphosphate (ATP), nigericin, SiO_2_, poly (deoxyadenylic-thymidylic) acid sodium salt (poly (dA:dT)), polyinosinic: polycytidylic acid (poly (I:C)), Pam3CSK4, dimethyl sulfoxide (DMSO) and LPS (*Escherichia coli*, 055: B5) were purchased from Sigma-Aldrich (Munich, Germany). Epimedin A (110623–72-8, purity 99.0%), epimedin A_1_ (140147–77-9, purity 99.92%), epimedin B (110623–73-9, purity 99.39%), epimedin C (110642–44-9, purity 99.1%), icariin (489–32-7, purity 97.64%), icaritin (118525–40-9, purity 99%), Icariside I (56725–99-6, purity 99.47%) and anhydroicaritin (38226–86-7, purity 99.51%) were purchased from TargetMol. *Salmonella* was kindly provided by Dr. Tao Li from National Center of Biomedical Analysis. MCC950 was obtained from TargetMol (Boston, USA). Anti-mouse caspase-1(1:1000, AG-20B-0042) was purchased from Adipogen (San Diego, USA). anti-mouse IL-1β (1:1000, 12,507), and anti-NLRP3 (1:2000, 15101S) were obtained from Cell Signaling Technology (Boston, USA). Anti-ASC (1:1000, sc-22,514-R) was purchased from Santa Cruz Biotechnology (Dallas, USA). Anti-GAPDH (1:2000, 60,004–1-1 g) was purchased from Proteintech (Chicago, USA). Color Prestained Protein marker (20AB01) was purchased from GenStar (Beijing, China).

### Cell culture

Bone-marrow-derived macrophages (BMDMs) were isolated from the femoral bone marrow of 10-week-old female C57BL/6 mice and cultured in Dulbecco’s modified Eagle’s medium (DMEM) complemented with 10% fetal bovine serum (FBS), 1% penicillin/streptomycin (P/S) and 50 ng/mL murine macrophage colony-stimulating factor (M-CSF). All cell lines were cultured under a humidified 5% (v/v) CO_2_ atmosphere at 37 °C.

### Inflammasome activation

We seeded BMDMs at 4 × 10^5^ cells/well in 24-well plates overnight. The following day, the medium was replaced, and cells were stimulated with 50 ng/mL LPS for 4 h. Next, the main components from EF were given for 1 h. The method for inflammasomes activation has been described previously [24].

### Western blotting

The method of protein extraction and western blotting assay on cell culture supernatant and whole cell lysis have been described previously [24].

### Caspase-1 activity assay

The Caspase-Glo® 1 Inflammasome Assay (Promega, Beijing, China) was used to assess caspase-1 activity in cell culture supernatant according to the manufacturer’s instructions.

### Enzyme-linked immunosorbent assay (ELISA)

ELISA measurements of mouse IL-1β, TNF-α (Dakewe, Beijing, China) were made in accordance with the manufacturer’s directions.

### Alanine aminotransferase (ALT) and aspartate transaminase (AST)

Serum ALT and AST were determined using the commercially available assay kit (Nanjing Jiancheng Bioengineering Institute, Nanjing, China) according to the manufacturer’s instructions.

### Lactate dehydrogenase (LDH) assay

LPS-primed BMDMs were treated with inflammasome stimulants in the presence of Icariside I. The release of LDH into the culture supernatant was determined by LDH cytotoxicity assay kit (Beyotime, Shanghai, China) according to the manufacturer’s instructions.

### ASC oligomerization

The method for ASC oligomerization has been elucidated in prior studies [25].

### Intracellular potassium detection

The assay for intracellular potassium has been mentioned in a previous report [26].

### Confocal microscopy

Confocal microscopy analysis, which is carried out to test mitochondrial damage, has been described previously [26].

### Mitochondrial reactive oxygen species assay

BMDMs were put onto 100 mm diameter culture dishes and primed with LPS (50 ng/ml) for 4 h. Then, cells were detached and transferred into 1.5 ml tubes for 1 h Icariside I treatment. Then, cells were stimulated with ATP, nigericin or SiO_2_, after which the cells were washed twice with Hank’s balanced salt solution (HBSS). For mitochondrial ROS (mtROS) measurements, BMDMs were loaded with 4 μM MitoSOX red mitochondrial superoxide indicator (Invitrogen) (Ex/Em: 510/580 nm) for 20 min and washed twice with HBSS. After staining and washing, cells were resuspended in HBSS and flow cytometry was conducted to measure mtROS.

### LPS/Icariside I cotreatment-induced IDILI in vivo

Female 6–8-week-old C57BL/6 mice were given 2 mg/kg LPS or its saline vehicle iv via a tail vein. 2 h later, Icariside I (50 mg/kg) was administered through intraperitoneal injection. Mice serum and a fraction of liver samples were collected 6 h after Icariside I administration, and a portion of each excised liver was fixed in 10% formalin neutral buffer solution and used for immunohistochemical staining. The degree of liver injury was assessed by histopathological staining with hematoxylin and eosin (H&E) staining and CD45, CD64, Ly6G immunohistochemistry, the serum IL-1β, TNF-α, ALT and AST levels, the serum leucocyte infiltration by FACS. Moreover, the caspase-1 activity in the liver homogenate was measured and normalized to the total protein level using a BCA protein quantification kit (Solarbio, Beijing, China) according to the manufacturer’s instructions.

### MCC950 blocks Icariside I-induced IDILI in vivo

Female 6–8-week-old C57BL/6 mice were given 50 mg/kg MCC950 or its saline vehicle through intraperitoneal injection. 1 h later, 2 mg/kg LPS or its saline vehicle was given iv via a tail vein. Then, 2 h later, Icariside I (50 mg/kg) was administered through intraperitoneal injection. Mice serum and a fraction of liver samples were collected after 6 h. Liver injury was characterized through histopathological staining with hematoxylin and eosin (H&E), CD45, CD64, Ly6G immunohistochemistry, the serum levels of IL-1β, TNF-α, ALT and AST, the serum leucocyte infiltration by FACS and the activity of caspase-1 in the liver homogenate as mentioned previously.

### Statistical analyses

The software Prism 6 and SPSS statistics 21.0 were used for statistics and analysis. All experimental data were expressed as the means ± Standard Error of Mean (SEM). A two-tailed unpaired Student’s t-test was conducted to evaluate the significant differences in two groups. *P* < 0.05 was considered significant.

## Results

### Icariside I enhances NLRP3 inflammasome activation triggered by ATP and nigericin, but not SiO_2_, poly(I:C) and cytosolic LPS

**Fig. 1:**
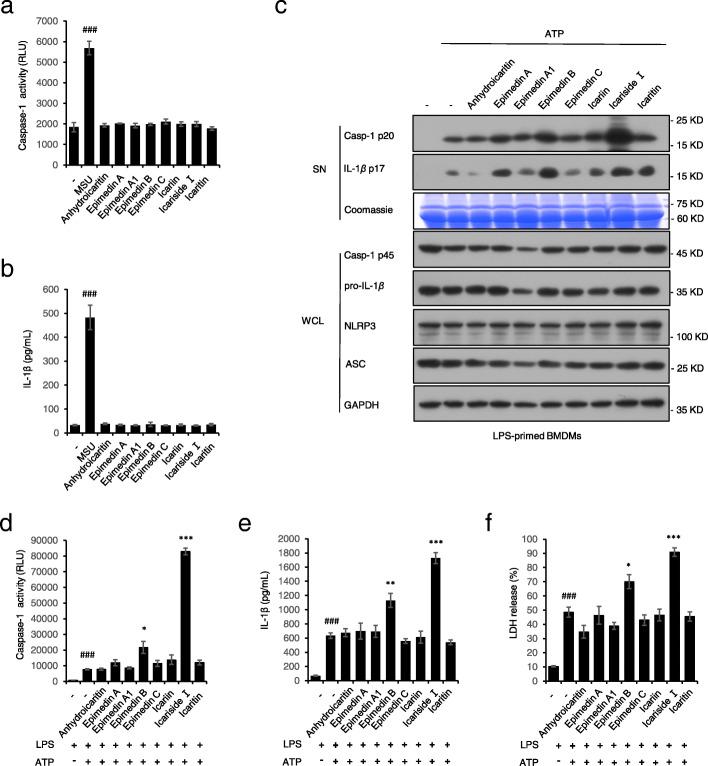
Effect of the main compositions from EF on NLRP3 inflammasome activation. **a**, **b** Caspase-1 activity (**a**) and ELISA of IL-1β (**b**) in supernatants (SN) from LPS-primed BMDMs treated with MSU (200 μg/ml) or the main compositions from EF for 4 h. **c** Western blots of SN and whole cell lysates (WCL) from LPS-primed BMDMs treated with the main compositions from EF(20 μM) and then stimulated with ATP(5 mol/L). **d**-**f** Caspase-1 activity (**d**), ELISA of IL-1β (**e**) and the release of LDH (**f**) in SN from LPS-primed BMDMs treated with the main compositions from EF (20 μM) and then stimulated with ATP. RLUs, the relative light units. Data are represented as the mean ± SD from at least three biological samples. ^##^*p* < 0.01,^###^*p* < 0.001 vs. the control group. *P < 0.05, **P < 0.01, ***P < 0.001 vs. the LPS plus ATP group

Eight compounds (epimedin A, epimedin A1, epimedin B, epimedin C, icariin, icaritin, Icariside I and anhydroicaritin) derived from EF were chosen to test whether the ingredients in EF could activate the NLRP3 inflammasome. None of them induced NLRP3 inflammasome activation as an agonist (Fig. 1a, b), but Icariside I and epmedin B significantly promoted caspase-1 activation and IL-1β production induced by ATP in LPS-primed BMDMs (Fig. C-F). In particular, Icariside I, which has the most potent effect on NLRP3 inflammasome activation, may be the main component contributing to the EF-induced liver injury. Therefore, we next investigated the effect and mechanism of Icariside I on NLRP3 inflammasome activation.

**Fig. 2:**
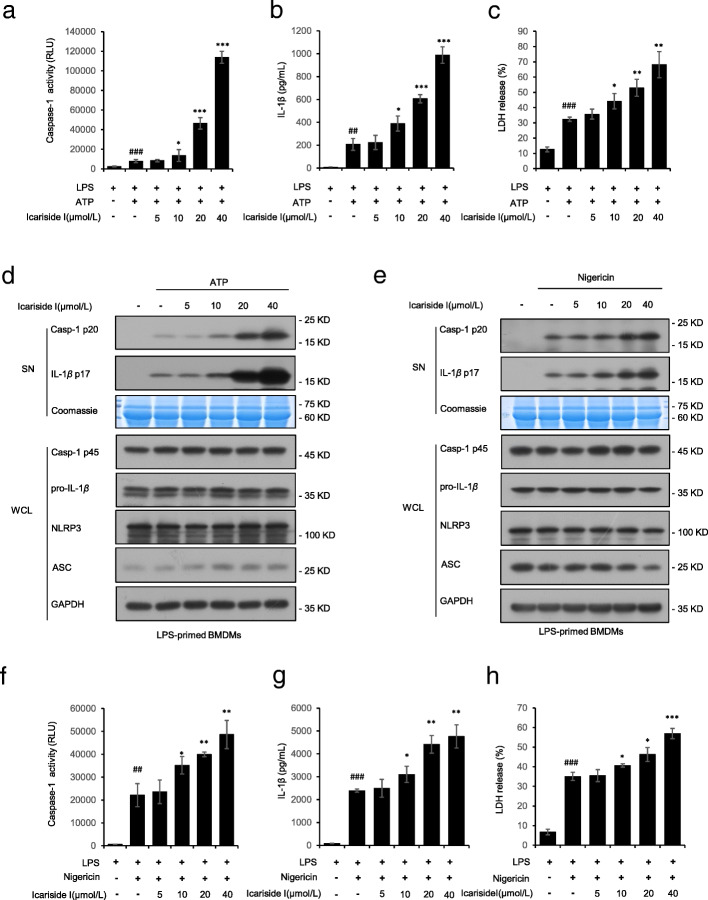
Icariside I promotes NLRP3 inflammasome activation stimulated by ATP and nigericin. **a**-**c** Caspase-1 activity (**a**) and ELISA of IL-1β (**b**), the release of LDH (**c**) in SN from LPS-primed BMDMs treated with various doses of Icariside I and then stimulated with ATP. (**d**) Western blots of SN and WCL  from LPS-primed BMDMs treated with various doses of Icariside I and then stimulated with ATP. **e** Western blots of SN and WCL from LPS-primed BMDMs treated with various doses of Icariside I before nigericin stimulation. **f**-**h** Caspase-1 activity (**f**), ELISA of IL-1β (**g**) and the release of LDH (**h**) from LPS-primed BMDMs treated with various doses of Icariside I and then stimulated with nigericin. RLUs, the relative light units. Data are represented as the mean ± SD from at least three biological samples. ^##^*P* < 0.01, ^###^*p* < 0.001 vs. the control group. *P < 0.05, **P < 0.01, ***P < 0.001 vs. the LPS plus ATP or nigericin group

Next, we probe into the effect of Icariside I on NLRP3 inflammasome activation. BMDMs were first primed with LPS, then pretreated with Icariside I and finally stimulated with the NLRP3 stimulus ATP. The results showed that Icariside I treatment increased the caspase-1 cleavage, and IL-1β maturation induced by ATP in a dose-dependent manner (Fig. 2a, b). In addition, the release of lactate dehydrogenase (LDH) induced by ATP in LPS-primed BMDMs was significantly promoted by Icariside I (Fig. 2c), suggesting that Icariside I increases caspase-1-mediated pyroptosis. In addition, the expression of NLRP3, ASC, pro-IL-1β, and pro-caspase-1 (p45) in cell lysates was not affected by Icariside I (Fig. 2d). Next, we also assessed the impact of Icariside I on nigericin-induced NLRP3 inflammasome activation in BMDMs. The results showed that Icariside I treatment increased production of the caspase-1 and IL-1β and the release of LDH triggered by nigericin in LPS-primed BMDMs (Fig. 2f-h) in a dose-dependent manner. However, Icariside I treatment did not affect the expression of pro-IL-1β and pro-caspase-1 in cell lysates (Fig. 2e).

**Fig. 3:**
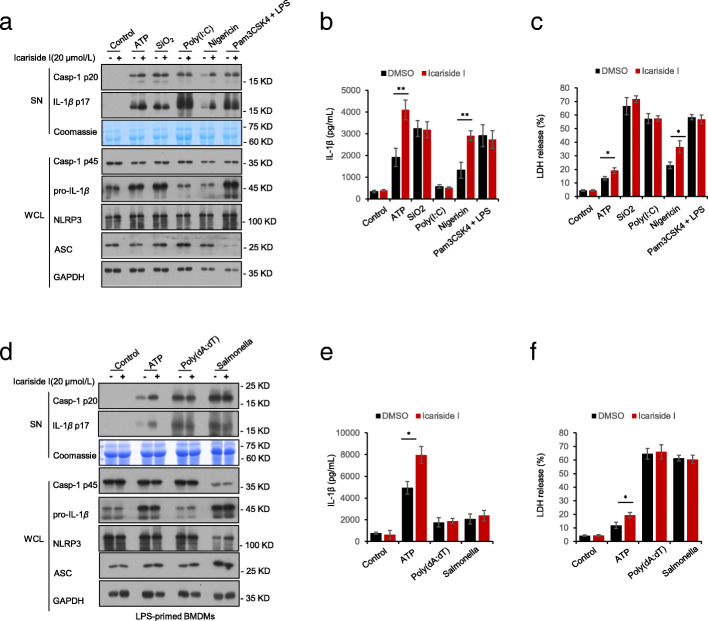
Icariside I has no effect on other stimuli-induced NLRP3 inflammasome activation and also fails to upregulate the activation of non-canonical NLRP3 inflammasome, as well as AIM2 and NLRC4 inflammasomes. **a**, **d** Western blot analysis of caspase-1 (p20) and IL-1β in SN of LPS-primed BMDMs treated Icariside I (20 μM) and then stimulated with ATP, nigericin, poly(I:C), SiO2, poly (dA:dT), and Salmonella or Pam3CSK4-primed BMDMs treated with Icariside I (20 μM) and then stimulated with LPS, and Western blot analysis of pro- IL-1β, caspase-1 (p45), NLRP3 and ASC in WCL. **b**-**c**, **e**-**f** ELISA of IL-1β (**b**, **e**) and the release of LDH (**c**, **f**) in SN. RLUs, the relative light units. Coomassie blue staining was used as the loading control in the SN **a**, **d**.  Data are represented as the mean ± SD from at least three biological samples. The significance of the differences was analyzed using an unpaired Student’s t-test: *P < 0.05, **P < 0.01 vs. the control

We also tested the effect of Icariside I on the other stimuli-induced NLRP3 inflammasome activation. Our results showed that pretreatment with Icariside I had no effect on caspase-1 cleavage, IL-1β secretion and LDH release triggered by SiO_2_ and poly(I:C) (Fig. 3a-c). Additionally, the noncanonical NLRP3 inflammasome is activated by intracellular LPS. We tested cytosolic LPS-induced noncanonical NLRP3 inflammasome activation with or without Icariside I treatment, and the results also showed that Icariside I did not alter caspase-1 cleavage, IL-1β secretion, and LDH release in Pam3CSK4-primed BMDMs transfected with LPS (Fig. 3a-c). Also, Icariside I treatment did not affect the expression of pro-IL-1β, ASC, NLRP3 and pro-caspase-1(Fig. 3a) in cell lysate. These results suggest that Icariside I specifically enhances NLRP3 inflammasome activation triggered by ATP and nigericin, but not SiO_2_, poly(I:C) and cytosolic LPS.

### Icariside I has no effect on NLRC4 and AIM2 inflammasomes activation

We also examined whether Icariside I could promote the activation of NLRC4 and AIM2 inflammasomes, which can be activated by *Salmonella typhimurium* infection and poly (dA:dT) transfection, respectively. LPS-primed BMDMs were infected with *Salmonella typhimurium* to activate NLRC4 inflammasomes, and the results indicated that Icariside I did not alter the release of IL-1β and LDH in response to *Salmonella typhimurium* infection (Fig. 3d-f). The effect of Icariside I on AIM2 inflammasome was examined by transfecting LPS-primed BMDMs with poly (dA: dT). The results also showed that Icariside I did not enhance caspase-1 cleavage, IL-1β secretion and LDH release triggered by poly (dA:dT) transfection (Fig. 3d-f). The expression of NLRP3, ASC, pro-IL-1β and pro-caspase-1 (p45) in cell lysate was also not affected by Icariside I treatment (Fig. 3d). Taken together, these results demonstrated that Icariside I has no effect on NLRC4 and AIM2 inflammasomes activation.

### Icariside I promotes ATP or nigericin-induced ASC oligomerization but has no effect on K^+^ efflux

**Fig. 4:**
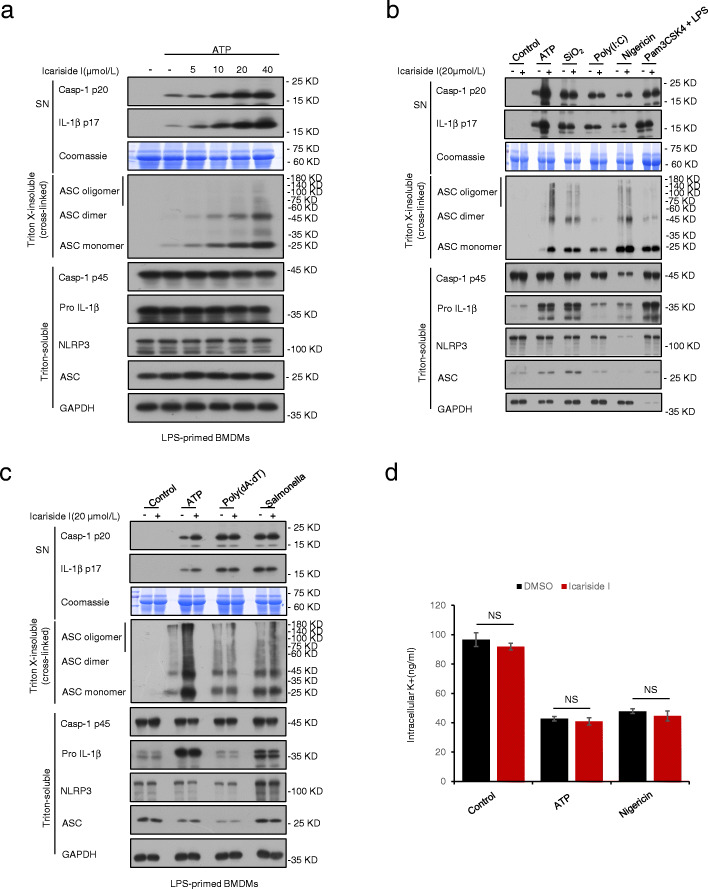
Icariside I promotes ATP or nigericin-induced ASC oligomerization but has no effect on K^+^ efflux. **a** Western blot analysis of ASC oligomerization in cell lysates of LPS-primed BMDMs treated with various doses of Icariside I and then stimulated with ATP. **b**, **c** Western blot analysis of ASC oligomerization in cell lysates of LPS-primed BMDMs treated with Icariside I (20 μM) and then stimulated with ATP, nigericin, poly(I:C), SiO_2_, poly (dA:dT), *Salmonella* or Pam3CSK4-primed BMDMs treated with Icariside I (20 μM) and then stimulated with LPS.  Data are represented from at least three biological samples.﻿ **d** Qualification of potassium efflux in LPS-primed BMDMs treated with Icariside I (20 μM) and then stimulated with ATP and nigericin. Data are represented as the mean ± SD from at least three biological samples.﻿ NS, not significant

During NLRP3 inflammasome activation, ASC oligomerization is a critical step for caspase-1 activation. Therefore, we investigated whether Icariside I could regulate ASC oligomer formation. Immunoblot analysis was used to test ASC oligomerization in LPS-primed BMDMs treated or untreated with Icariside I and then stimulated with NLRP3 inflammasome stimuli. Upon NLRP3 inflammasome activation by stimuli, cytosolic fractions from cell lysates were cross-linked. Consistent with the effect of Icariside I on the production of caspase-1 and IL-1β, Icariside I dose-dependently promoted ATP-induced ASC oligomerization in LPS-primed BMDMs (Fig. 4a). Similarly, we also observed that Icariside I promoted ASC oligomerization and the production of caspase-1 and IL-1β induced by nigericin but not SiO2, poly(I:C) and intracellular LPS (Fig. 4b). Moreover, ASC oligomerization induced by Salmonella typhimurium and poly (dA: dT) was not affected by Icariside I (Fig. 4c). These results suggest that Icariside I specifically enhances ATP or nigericin-induced NLRP3 inflammasome activation. However, ASC oligomerization is necessary for all agonist-induced NLRP3 inflammasome activation, so we speculate that Icariside I does not directly target ASC to exacerbate ATP or nigericin-induced NLRP3 inflammasome activation. Overall, these results indicated that Icariside I may act on upstream events of ASC oligomerization to exacerbate ATP or nigericin-induced NLRP3 inflammasome activation. Next, we sought to study whether Icariside I affects K+ efflux in NLRP3 inflammasome activation. The result showed that Icariside I promoted the release of IL-1β induced by ATP and nigericin in a dose-dependent manner, but it has no effect on K+ efflux triggered by these stimuli (Fig. 4d), suggesting K+ efflux does not contribute to the enhancement effect of Icariside I on ATP or nigericin-induced NLRP3 inflammasome activation. NEK7 is involved in the ATP/nigericin-dependent induction of NLRP3 without affecting K^+^ influx. Thus, we transfected HEK-293 T cells with plasmids expressing full-length Flag-tagged NLRP3 and then treated with Icariside I. The results showed that Icariside I treatment have no effect on the interaction of NEK7 with NLRP3 (Supplementary Fig. 2).

### Icariside I facilitates ATP or nigericin-induced NLRP3 inflammasome activation dependent on mitochondrial ROS production

**Fig. 5:**
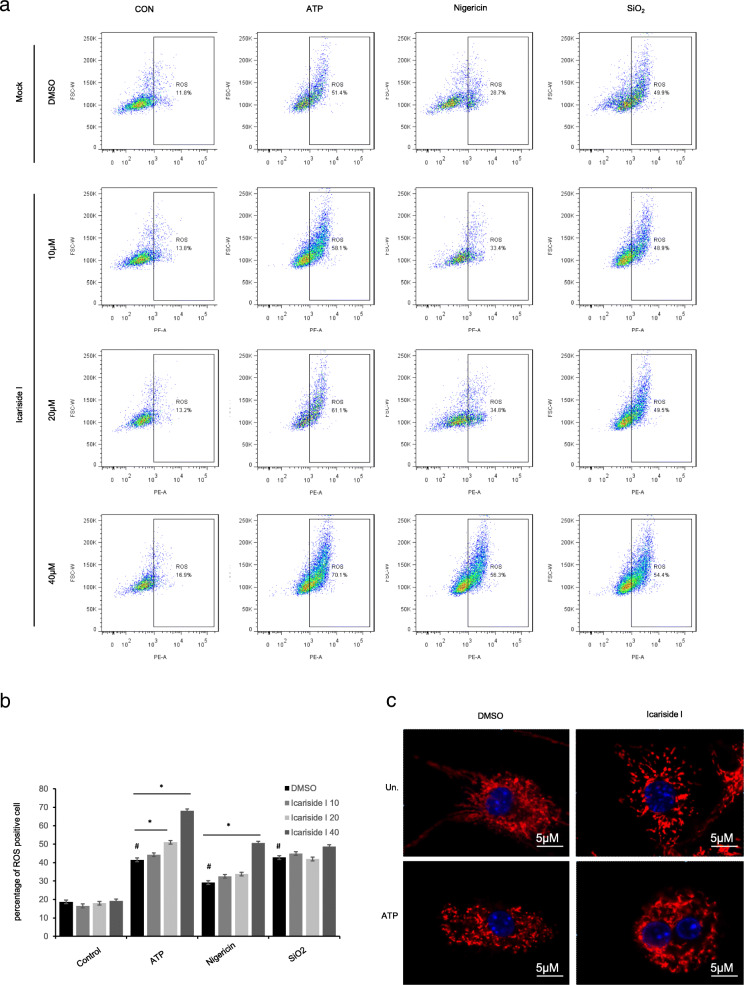
Icariside I facilitates ATP/nigericin-induced NLRP3 inflammasome activation dependent on mitochondrial ROS production. **a**, **b** LPS-primed BMDMs were treated with Icariside I before stimulated with ATP, nigericin, SiO2. BMDMs were loaded with MitoSOX red mitochondrial superoxide indicator (Ex/Em: 510/580 nm). After staining and washing, flow cytometry was conducted to test mtROS production. **c** Confocal microscopy analysis in LPS-primed BMDMs treated with Icariside I (20 μM) and then left stimulated with ATP, followed by staining with Mitotracker red and DAPI. Data are represented as the mean ± SD from at least three biological samples. ^#^*p* < 0.05 vs. the control group. *P < 0.05

We examined the effect of Icariside I on mitochondrial reactive oxygen species (ROS) production and mitochondrial dysfunction. As shown in Fig. 5c, ATP-induced mitochondrial damage was observed in BMDMs, but mitochondrial damage was not induced after Icariside I treatment alone. The MitoSOX Red Mitochondrial Superoxide indicator assay was used to quantify the amount of mtROS during the course of ATP, nigericin, SiO_2_ treatment in the presence or absence of Icariside I. The results showed that mtROS production was not induced after Icariside I treatment alone, but Icariside I successfully potentiated mtROS production induced by ATP and nigericin rather than SiO2 (Fig. 5a, b, Supplementary Fig. 2). Thus, these results indicate that synergistic induction of ROS production is a crucial event in the enhancement effect of Icariside I on NLRP3 inflammasome triggered by ATP and nigericin. Next, added a ROS scavenger N-acetylcysteine (NAC), which is an inhibitor of mitochondrial ROS production, to evaluate the ATP/nigericin-dependent activity of Icariside I on NLRP3 activation is mediated by ROS mitochondrial production. Results showed that NAC treatment suppressed mitochondrial ROS production (Supplementary Fig. 3). Most importantly, NAC treatment could reverse Icariside I -induced caspase-1 maturation and IL-1β production when stimulated with ATP (Supplementary Fig. 3). Therefore, sufficient evidences could be believed that Icariside I facilitates ATP/nigericin-induced NLRP3 inflammasome activation by increasing mitochondrial ROS production.

### The combination of LPS and Icariside I induces liver injury in WT mice but not in MCC950 pretreatment mice

**Fig. 6:**
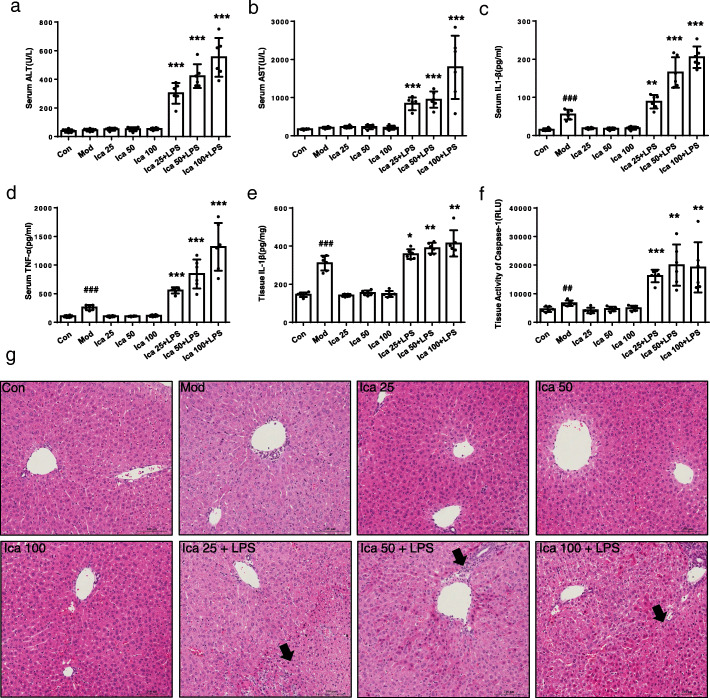
Early liver injury and inflammatory mediator production after Icariside I/LPS cotreatment. **a**-**f** WT female C57BL/6 mice were pretreated with LPS (2 mg/kg) through the tail vein. 2 h later, intraperitoneally Icariside I (25 mg/kg, 50 mg/kg, 100 mg/kg, *n* = 6) injection was conducted. 6 h after Icariside I injection, serum levels of ALT (**a**), AST (**b**), IL-1β (**c**), TNF-α (**d**) were measured by assay kit, (**e**, **f**) IL-1β (**e**) and caspase-1 activity (**f**) in the livers was detected after BCA protein quantification and normalization processing. **g** H&E staining. Data are expressed as the mean ± S. ^#^^*#*^*P* < 0.01, ^###^*P* < 0.001, vs. the control. *P < 0.05, **P < 0.01, ***P < 0.01

LPS can be recognized by toll-like receptors (TLRs), which leads to the stimulation of inflammatory cells and the consequent expression and release of numerous inflammatory mediators. We investigated the effects of Icariside I in an LPS-mediated susceptible mouse model of IDILI. As expected, the results showed that Icariside I dose-dependently induced the elevation of ALT and AST serum levels in the LPS-mediated mice model (Fig. 6a, b). In addition, Icariside I treatment also significantly increased the production of IL-1β and TNF-α production in vivo (Fig. 6c, d). Likewise, liver histology showed that Icariside I and LPS alone treatment did not alter liver tissue structure in mice, but the combination of LPS and Icariside I led to a trend of hepatocyte focal necrosis and inflammatory cell infiltration in the liver tissue of mice (Fig. 6g). We also assessed the leucocyte infiltration by FACS, the results showed that Icariside I facilitate the number of macrophages in the LPS-mediated mice model (Supplementary Fig. 4, 5, 6, 7). Moreover, the immunohistochemistry experiments showed that the combination of LPS and Icariside I facilitate the number of leucocytes, macrophages and neutrophils (Supplementary Fig. 8, 9, 10). Thus, these results indicate that Icariside I could induce liver injury in an LPS-mediated susceptibility mouse model of IDILI.

**Fig. 7:**
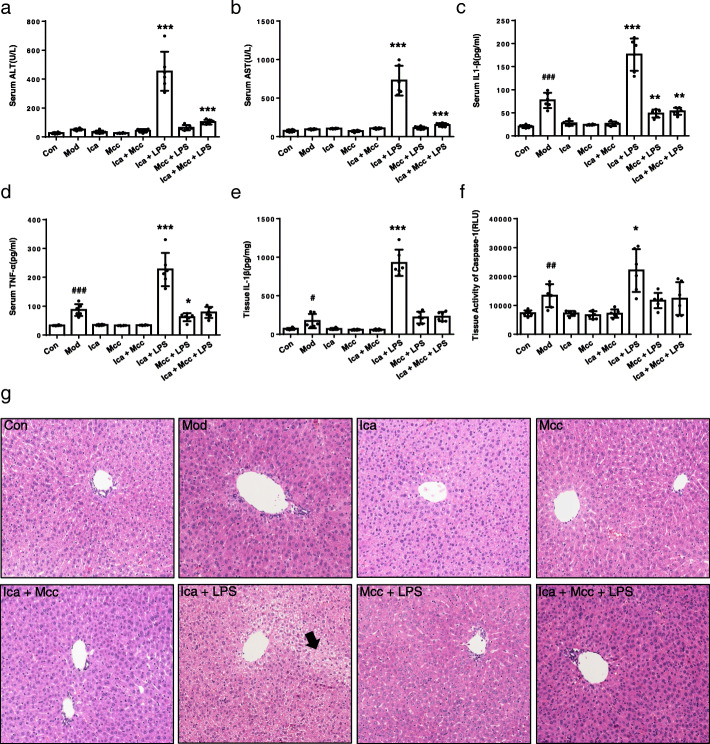
Icariside I/LPS induces liver injury in WT mice but not in NLRP3 inflammasome knockdown mice. **a**-**f**  WT female C57BL/6 mice were given 50 mg/kg MCC950 or its saline vehicle through intraperitoneal injection. Then, mice were pretreated with LPS (2 mg/kg) through the tail vein. 2 h later, intraperitoneally Icariside I (50 mg/kg *n* = 6) injection was conducted. 6 h after Icariside I injection, serum levels of ALT (**a**), AST (**b**), IL-1β (**c**), TNF-α (**d**) were measured by assay kit, (**e**, **f**) IL-1β (**e**) and caspase-1 activity (**f**) in the livers was detected after BCA protein quantification and normalization processing. **g** H&E staining. Data are expressed as the mean ± S. ^#^*P* < 0.05, ^##^*P* < 0.01, ^###^*P* < 0.001 vs. the control. *P < 0.05, **P < 0.01, ***P < 0.001

As reported, MCC950 is a specific small-molecule inhibitor of NLRP3 inflammasome, so administration of MCC950 was used to mimic the consequences of NLRP3 inflammasome-knockdown in mice [27]. Mice were injected intraperitoneally (i.p.) with MCC950 for 1 h, followed by LPS (i.v.) and Icariside I (i.p.) for 2 h, then assessed 6 h later. The results showed that pretreatment with MCC950 inhibited IL-1β and caspase-1 production in LPS-mediated mouse model, indicating that MCC950 is active in vivo (Fig. 7e, f). Icariside I treatment led to the elevation of serum levels of ALT, AST, IL-1β and TNF-α in LPS-mediated mouse model, but not in mice cotreated with LPS and MCC950 (Fig. 7a-d). Notably, histopathologic studies showed that Icariside I treatment induced hepatocyte focal necrosis and inflammation in LPS-mediated mouse model but not in other groups (Fig. 7g). Moreover, immunohistochemistry indicated that the combination of LPS and Icariside I facilitate the number of leucocytes, macrophages, and neutrophils (Supplementary Fig. 8, 9, 10). The leucocyte infiltration by FACS also showed that Icariside I facilitate the number of macrophages in the LPS-mediated mouse model (Supplementary Fig. 4, 5, 6, 7). These results suggest that the combination of LPS and Icariside I can induce liver injury in WT mice but not in MCC950 pretreatment mice. Taken together, these data clearly confirmed that Icariside I could induce idiosyncratic hepatotoxicity by promoting NLRP3 inflammasome activation in vivo.

## Discussion

IDILI only has rare adverse reactions in a small number of susceptible individuals, but it is one of the most common serious adverse reactions in clinical practice, as it can cause acute liver failure and even death [5, 28–31]. IDILI is often difficult to predict based solely on dose and pharmacological action and affects only susceptible individuals. Therefore, it is a challenging issue to elucidate the factors that mark susceptibility to IDILI. NLRP3 inflammasome is the primary sensor for inflammatory signals and is a key instigator of inflammatory reactions in a variety of diseases. Emerging evidence supports the central role of NLRP3 inflammasomes in the pathogenesis of many liver diseases, including alcoholic and nonalcoholic fatty liver disease as well as liver injury [19, 32–34]. Additionally, previous studies have indicated that some drugs such as amodiaquine and nevirapine with the ability to induce IDILI, could induce the release of DAMPs from hepatocytes, leading to NLRP3 inflammasome activation in macrophages [35]. Besides, some drugs such as thioacetamide and carbon tetrachloride also induced hepatic damage by activating NLRP3 inflammasome [36–38]. EF is a commonly used herbal medicine for invigorating the liver and kidney, but the potential hepatotoxicity of EF limits its clinical application. Our previous study indicated that EF could lead to IDILI, which has been confirmed by clinical cases and an LPS-mediated susceptibility mouse model of IDILI [14]. Meanwhile, our data indicate that IL-1β produced by NLRP3 inflammasome activation is a marker for EF-induced idiosyncratic hepatotoxicity. Icariside I is not only found in EF but is also one of the major metabolite of icariin, which is used as an indicator for the quality of EF based on the Chinese pharmacopoeia. Here, we demonstrate that Icariside I, one of the major active or metabolic constitutes of EF, facilitates ATP or nigericin-induced NLRP3 inflammasome activation in vitro and causes idiosyncratic hepatotoxicity by promoting NLRP3 inflammasome activation in vivo, suggesting that Icariside I contributes to EF-induced idiosyncratic hepatotoxicity through enhancing NLRP3 inflammasome activation.

In this study, we demonstrate that Icariside I could promote NLRP3 inflammasome activation triggered by ATP and nigericin but not SiO_2_, poly(I:C) and cytosolic LPS. Our results also showed that Icariside I does not affect the activation of the NLRC4 or AIM2 inflammasome. These data indicate that Icariside I could specifically enhance ATP or nigericin-induced NLRP3 inflammasome activation. In addition, Icariside I treatment also promotes ASC oligomerization, which is a key event in NLRP3 inflammasome activation. However, ASC oligomerization is necessary for all agonist-induced NLRP3 inflammasome activation, so we believe that Icariside I acts on the upstream signaling events of ASC oligomerization to exacerbate ATP or nigericin-induced NLRP3 inflammasome activation. K^+^ efflux is one of the main upstream events of NLRP3 inflammasome activation, but our study indicates that Icariside I does not alter K+ efflux triggered by ATP or nigericin, which suggests that Icariside I may target other upstream pathways or events of NLRP3 inflammasome activation. Mitochondrial damage and the release of mtROS are additional key upstream events of NLRP3 inflammasome activation. In this study, we found that Icariside I alone does not induce mitochondrial damage and mtROS production, but Icariside I could specifically amplify the production of mtROS triggered by ATP and nigericin but not by SiO_2_. Thus, these results demonstrated that Icariside I facilitates ATP or nigericin-induced NLRP3 inflammasome activation dependent upon mitochondrial ROS production.

Previous studies have shown that EF could induce liver injury and the production of IL-1β in the LPS-mediated susceptibility mouse model of IDILI. Icariside I is one of the main components of EF, so we evaluated whether Icariside I contributes to EF-induced idiosyncratic hepatotoxicity through enhancing NLRP3 inflammasome activation. Our data demonstrated that Icariside I could induce liver injury and promote the production of IL-1β in an LPS mouse model. MCC950, a specific inhibitor of NLRP3 inflammasome, was used to mimic the consequences of NLRP3 inflammasome knockdown in mice. The effect of Icariside I on liver damage is absent in the LPS-mediated mouse model pretreated with MCC950, and the results demonstrate that Icariside I could induce liver injury by promoting NLRP3 inflammasome activation in LPS-mediated susceptibility mouse model of IDILI. These results indicate that EF, Icariside I or its derivatives should be avoided in patients with ATP or nigericin-induced NLRP3 inflammasome activation-related diseases, which may be the risk factors for IDILI.

## Conclusions

In conclusion, the study illustrated that Icariside I exacerbates NLRP3 inflammasome activation triggered by ATP and nigericin. mtROS is a crucial contributor to the enhancing effect of Icariside I on ATP- or nigericin-induced NLRP3 inflammasome activation. The in vivo data demonstrated that the combination of non-hepatotoxic doses of LPS and Icariside I causes the increase of ALT, AST, IL-1β and TNF-α production, hepatocyte necrosis in WT mice, but not in mice cotreated with LPS and MCC950. The data suggests that Icariside I could cause idiosyncratic liver injury and may be a risk factor and responsible for EF-induced liver injury.

## Supplementary Information

**Additional file 1: Figure S1:** Icariside I facilitates ATP/nigericin-induced NLRP3 inflammasome activation dependent on mitochondrial ROS production. Flow cytometry was conducted to test mtROS production (two independent experiments). **Figure S2:** Icariside I doesn’t influence NEK7 interaction with NLRP3 in vitro. Immunoprecipitation (IP) and immunoblot analysis of the interaction of Flag-tagged NLRP3 in the lysates of HEK-293 T cells. Icariside I was added at 6 h post-transfection. **Figure S3:** Icariside I facilitates ATP/nigericin-induced NLRP3 inflammasome activation by increasing mitochondrial ROS production. (A, B) LPS-primed BMDMs were treated with Icariside I or NAC before stimulated with ATP. BMDMs were loaded with MitoSOX red mitochondrial superoxide indicator (Ex/Em: 510/580 nm). After staining and washing, flow cytometry was conducted to test mtROS production. (C) Western blots of SN and WCL from LPS-primed BMDMs treated with Icariside I, NAC or Icariside I plus NAC before ATP stimulation. (D) Caspase-1 activity in SN from LPS-primed BMDMs treated with Icariside I, NAC or Icariside I plus NAC and then stimulated with ATP. **Figure S4:** The leucocytes production after Icariside I/LPS cotreatment. Assessed the B cells infiltration by FACS. **Figure S5:** The leucocytes production after Icariside I/LPS cotreatment. Assessed the macrophages cells infiltration by FACS. **Figure S6:** The leucocytes production after Icariside I/LPS cotreatment. Assessed the neutrophils and infiltrating monocyte cells infiltration by FACS. **Figure S7:** The leucocytes production after Icariside I/LPS cotreatment. Assessed the T cells infiltration by FACS. **Figure S8:** The leucocytes production after Icariside I/LPS cotreatment. Immunohistochemistry experiments in tissue sections (CD45). **Figure S9:** The leucocytes production after Icariside I/LPS cotreatment. Immunohistochemistry experiments in tissue sections (Ly6G). **Figure S10:** The leucocytes production after Icariside I/LPS cotreatment. Immunohistochemistry experiments in tissue sections (CD64).

## Data Availability

Experimental data sets used and analysed during the current study as well as materials prepared are available from the corresponding author on reasonable request.

## Abbreviations

EF: Epimedii Folium
IDILI: Idiosyncratic drug-induced liver injury
ATP: Adenosine triphosphate
IL-1β: Interleukin 1β
mtROS: Mitochondrial reactive oxygen species
LPS: Lipopolysaccharide
TNF-α: Tumor necrosis factor-α
HDS: Herbal and dietary supplements
TCM: Traditional Chinese medicine
NLRP3: The nucleotide binding domain and leucine-rich repeat (NLR) pyrin domain containing 3
ASC: Apoptosis-associated speck-like protein containing CARD
caspase-1: Cysteinyl aspartate-specific proteinase-1
PAMPs: Pathogen-associated molecular patterns
DAMPs: Danger-associated molecular patterns
poly (dA:dT): Poly (deoxyadenylic-thymidylic) acid sodium salt
poly (I:C): Polyinosinic: polycytidylic acid
DMSO: Dimethyl sulfoxide
BMDMs: Bone-marrow-derived macrophages
DMEM: Dulbecco’s modified Eagle’s medium
FBS: Fetal bovine serum
P/S: Penicillin/streptomycin
M-CSF: Murine macrophage colony-stimulating factor
ALT: Alanine aminotransferase
AST: Aspartate Transaminase
LDH: Actate dehydrogenase
HBSS: Hank’s balanced salt solution
H&E: Histopathological staining with hematoxylin and eosin
TLRs: Toll-like receptors
i.p.: Intraperitoneally
MSU: Monocrystalline sodium urate
ELISA: Enzyme-linked immunosorbent assay

## References

[1] Fontana RJ (2014). Pathogenesis of idiosyncratic drug-induced liver injury and clinical perspectives. Gastroenterology.

[2] Uetrecht J (2019). Mechanistic Studies of Idiosyncratic DILI: Clinical Implications. Front Pharmacol.

[3] Uetrecht J (2019). Mechanisms of idiosyncratic drug-induced liver injury. Adv Pharmacol.

[4] Roth RA, Ganey PE (2011). Animal models of idiosyncratic drug-induced liver injury--current status. Crit Rev Toxicol.

[5] Kullak-Ublick GA, Andrade RJ, Merz M, End P, Benesic A (2017). Drug-induced liver injury: recent advances in diagnosis and risk assessment. Gut.

[6] Cho T, Uetrecht J (2017). How Reactive Metabolites Induce an Immune Response That Sometimes Leads to an Idiosyncratic Drug Reaction. Chem Res Toxicol.

[7] Shaw PJ, Beggs KM, Sparkenbaugh EM, Dugan CM, Ganey PE, Roth RA (2009). Trovafloxacin enhances TNF-induced inflammatory stress and cell death signaling and reduces TNF clearance in a murine model of idiosyncratic hepatotoxicity. Toxicol Sci.

[8] Poulsen KL, Olivero-Verbel J (2014). Trovafloxacin enhances lipopolysaccharide-stimulated production of tumor necrosis factor-alpha by macrophages: role of the DNA damage response. J Pharmacol Exp Ther.

[9] Gandhi A, Guo T, Shah P, Moorthy B, Ghose R (2013). Chlorpromazine-induced hepatotoxicity during inflammation is mediated by TIRAP-dependent signaling pathway in mice. Toxicol Appl Pharmacol.

[10] Metushi IG, Hayes MA, Uetrecht J (2015). Treatment of PD-1(−/−) mice with amodiaquine and anti-CTLA4 leads to liver injury similar to idiosyncratic liver injury in patients. Hepatology..

[11] Shen T, Liu Y, Shang J, Xie Q, Li J, Yan M (2019). Incidence and Etiology of Drug-Induced Liver Injury in Mainland China. Gastroenterology.

[12] Lin L, Ni B, Lin H, Zhang M, Li X, Yin X, Qu C, Ni J (2015). Traditional usages, botany, phytochemistry, pharmacology and toxicology of *Polygonum multiflorum* Thunb.: a review. J Ethnopharmacol.

[13] Tu C, He Q, Li CY, Niu M, Han ZX, Ge FL (2019). Susceptibility-Related Factor and Biomarkers of Dietary Supplement *Polygonum multiflorum*-Induced Liver Injury in Rats. Front Pharmacol.

[14] Gao Y, Wang Z, Tang J, Liu X, Shi W, Qin N (2020). New incompatible pair of TCM: Epimedii Folium combined with Psoraleae Fructus inducesidiosyncratic hepatotoxicity under immunological stress conditions. Front Med..

[15] Wang L, Li Z, Li L, Li Y, Yu M, Zhou Y, Lv X, Arai H, Xu Y (2014). Acute and sub-chronic oral toxicity profiles of the aqueous extract of Cortex Dictamni in mice and rats. J Ethnopharmacol.

[16] Schroder K, Tschopp J (2010). The inflammasomes. Cell.

[17] Elliott EI, Sutterwala FS (2015). Initiation and perpetuation of NLRP3 inflammasome activation and assembly. Immunol Rev.

[18] Wu X, Dong L, Lin X, Li J (2017). Relevance of the NLRP3 Inflammasome in the Pathogenesis of Chronic Liver Disease. Front Immunol.

[19] Szabo G, Csak T (2012). Inflammasomes in liver diseases. J Hepatol.

[20] Wen H, Ting JP, O'Neill LA (2012). A role for the NLRP3 inflammasome in metabolic diseases--did Warburg miss inflammation?. Nat Immunol.

[21] Ma H, He X, Yang Y, Li M, Hao D, Jia Z (2011). The genus Epimedium: an ethnopharmacological and phytochemical review. J Ethnopharmacol.

[22] Zhang L, Wang T, Zhao BS, Zhang JX, Yang S, Fan CL, Li P (2019). Effect of 2″-O-Rhamnosyl Icariside II, Baohuoside I and Baohuoside II in Herba Epimedii on Cytotoxicity Indices in HL-7702 and HepG2 Cells. Molecules..

[23] Zhong R, Chen Y, Ling J, Xia Z, Zhan Y, Sun E, Shi Z, Feng L (2019). The Toxicity and Metabolism Properties of Herba Epimedii Flavonoids on Laval and Adult Zebrafish. Evid Based Complement Alternat Med.

[24] Wang Z, Xu G, Gao Y, Zhan X, Qin N, Fu S (2019). Cardamonin from a medicinal herb protects against LPS-induced septic shock by suppressing NLRP3 inflammasome. Acta Pharm Sin B.

[25] Song N, Liu ZS, Xue W, Bai ZF, Wang QY, Dai J (2017). NLRP3 Phosphorylation Is an Essential Priming Event for Inflammasome Activation. Mol Cell.

[26] He H, Jiang H, Chen Y, Ye J, Wang A, Wang C (2018). Oridonin is a covalent NLRP3 inhibitor with strong anti-inflammasome activity. Nat Commun.

[27] Coll RC, Robertson AA, Chae JJ, Higgins SC, Munoz-Planillo R, Inserra MC (2015). A small-molecule inhibitor of the NLRP3 inflammasome for the treatment of inflammatory diseases. Nat Med.

[28] Chen M, Suzuki A, Borlak J, Andrade RJ, Lucena MI (2015). Drug-induced liver injury: Interactions between drug properties and host factors. J Hepatol.

[29] Chalasani NP, Hayashi PH, Bonkovsky HL, Navarro VJ, Lee WM, Fontana RJ (2014). ACG Clinical Guideline: the diagnosis and management of idiosyncratic drug-induced liver injury. Am J Gastroenterol.

[30] Navarro VJ, Khan I, Bjornsson E, Seeff LB, Serrano J, Hoofnagle JH (2017). Liver injury from herbal and dietary supplements. Hepatology..

[31] Roth AD, Lee MY (2017). Idiosyncratic Drug-Induced Liver Injury (IDILI): Potential Mechanisms and Predictive Assays. Biomed Res Int.

[32] Wree A, McGeough MD, Pena CA, Schlattjan M, Li H, Inzaugarat ME, Messer K, Canbay A, Hoffman HM, Feldstein AE (2014). NLRP3 inflammasome activation is required for fibrosis development in NAFLD. J Mol Med (Berl).

[33] Petrasek J, Bala S, Csak T, Lippai D, Kodys K, Menashy V, Barrieau M, Min SY, Kurt-Jones EA, Szabo G (2012). IL-1 receptor antagonist ameliorates inflammasome-dependent alcoholic steatohepatitis in mice. J Clin Invest.

[34] Dixon LJ, Flask CA, Papouchado BG, Feldstein AE, Nagy LE (2013). Caspase-1 as a central regulator of high fat diet-induced non-alcoholic steatohepatitis. PLoS One.

[35] Kato R, Uetrecht J (2017). Supernatant from Hepatocyte Cultures with Drugs That Cause Idiosyncratic Liver Injury Activates Macrophage Inflammasomes. Chem Res Toxicol.

[36] Dwivedi DK, Jena GB (2020). Diethylnitrosamine and thioacetamide-induced hepatic damage and early carcinogenesis in rats: Role of Nrf2 activator dimethyl fumarate and NLRP3 inhibitor glibenclamide. Biochem Biophys Res Commun.

[37] Mostafa ME, Shaaban AA, Salem HA (2019). Dimethylfumarate ameliorates hepatic injury and fibrosis induced by carbon tetrachloride. Chem Biol Interact.

[38] Dwivedi DK, Jena GB (2018). Glibenclamide protects against thioacetamide-induced hepatic damage in Wistar rat: investigation on NLRP3, MMP-2, and stellate cell activation. Naunyn Schmiedeberg's Arch Pharmacol.

