# Supplementary figures and images for: Icariside I specifically facilitates ATP or nigericin-induced NLRP3 inflammasome activation and causes idiosyncratic hepatotoxicity
Source: Cell Commun Signal. 2021 Feb 11;19:13. doi: 10.1186/s12964-020-00647-1 (PMC7879676; doi:10.1186/s12964-020-00647-1)

Figure S1

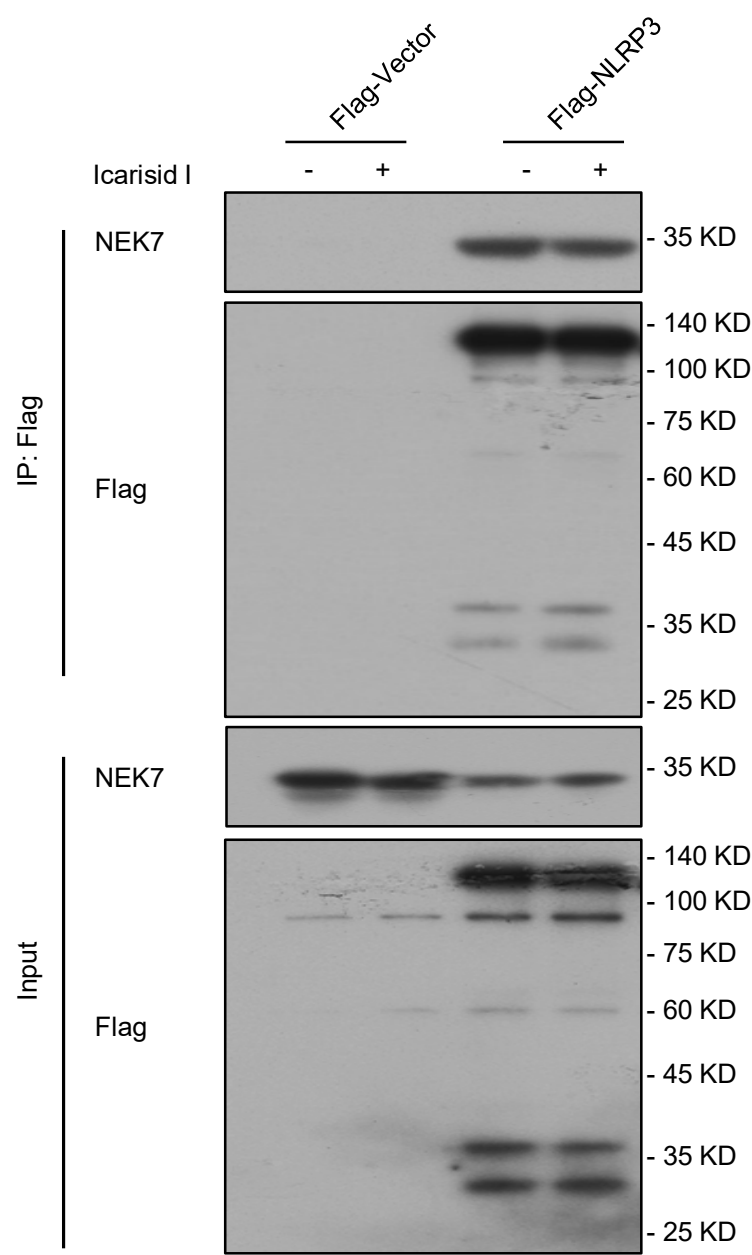

Figure S2

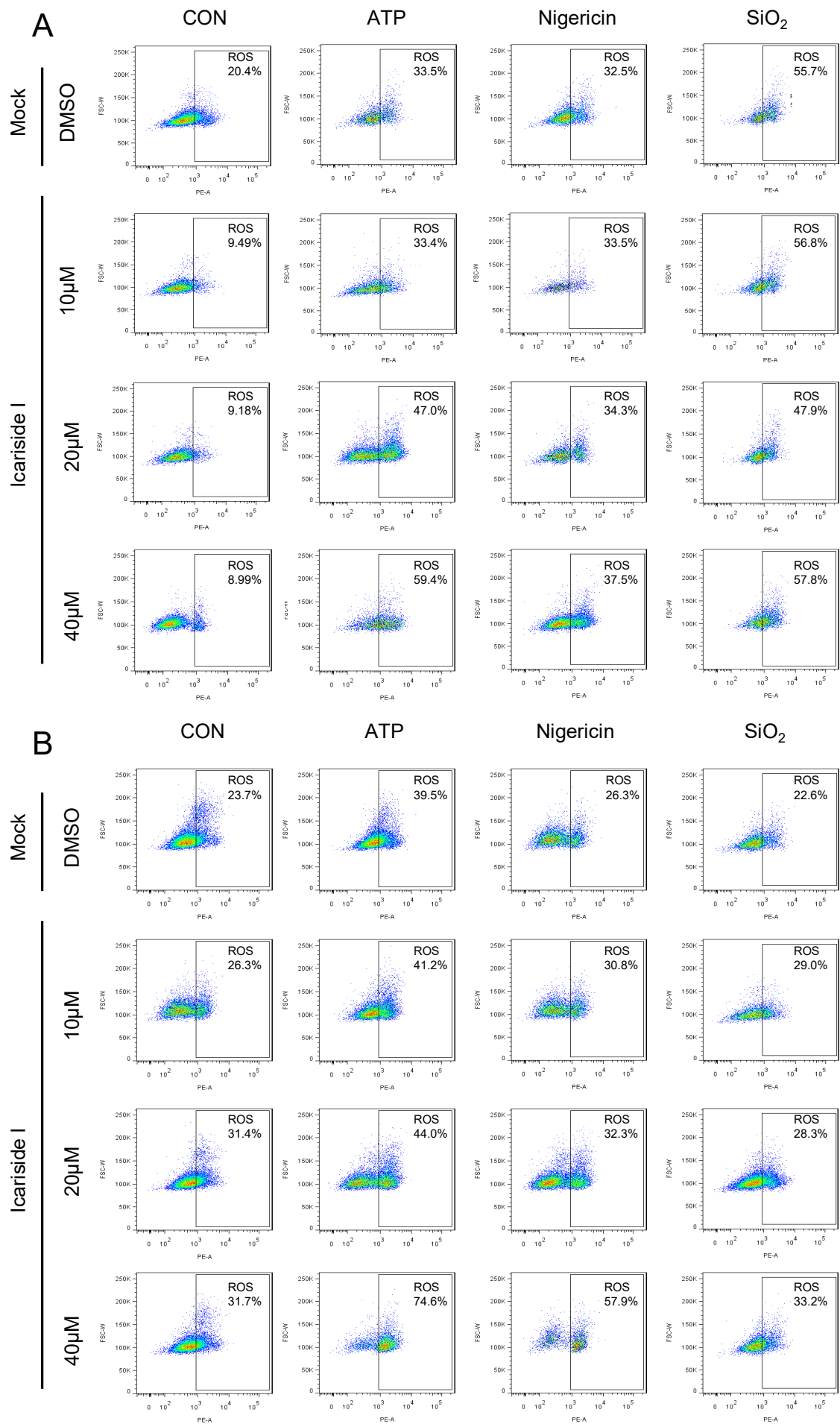

Figure S3

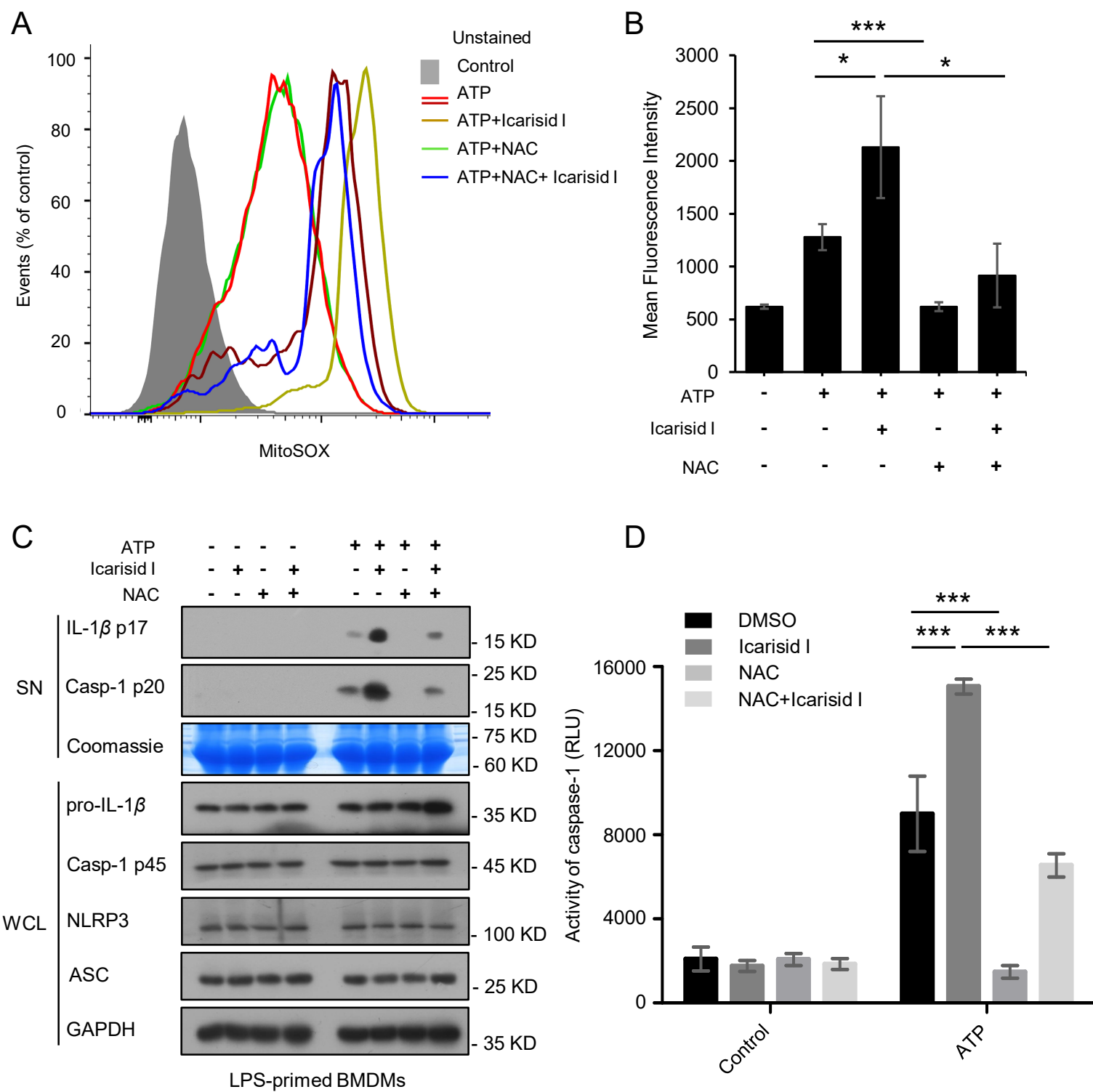

Figure S4

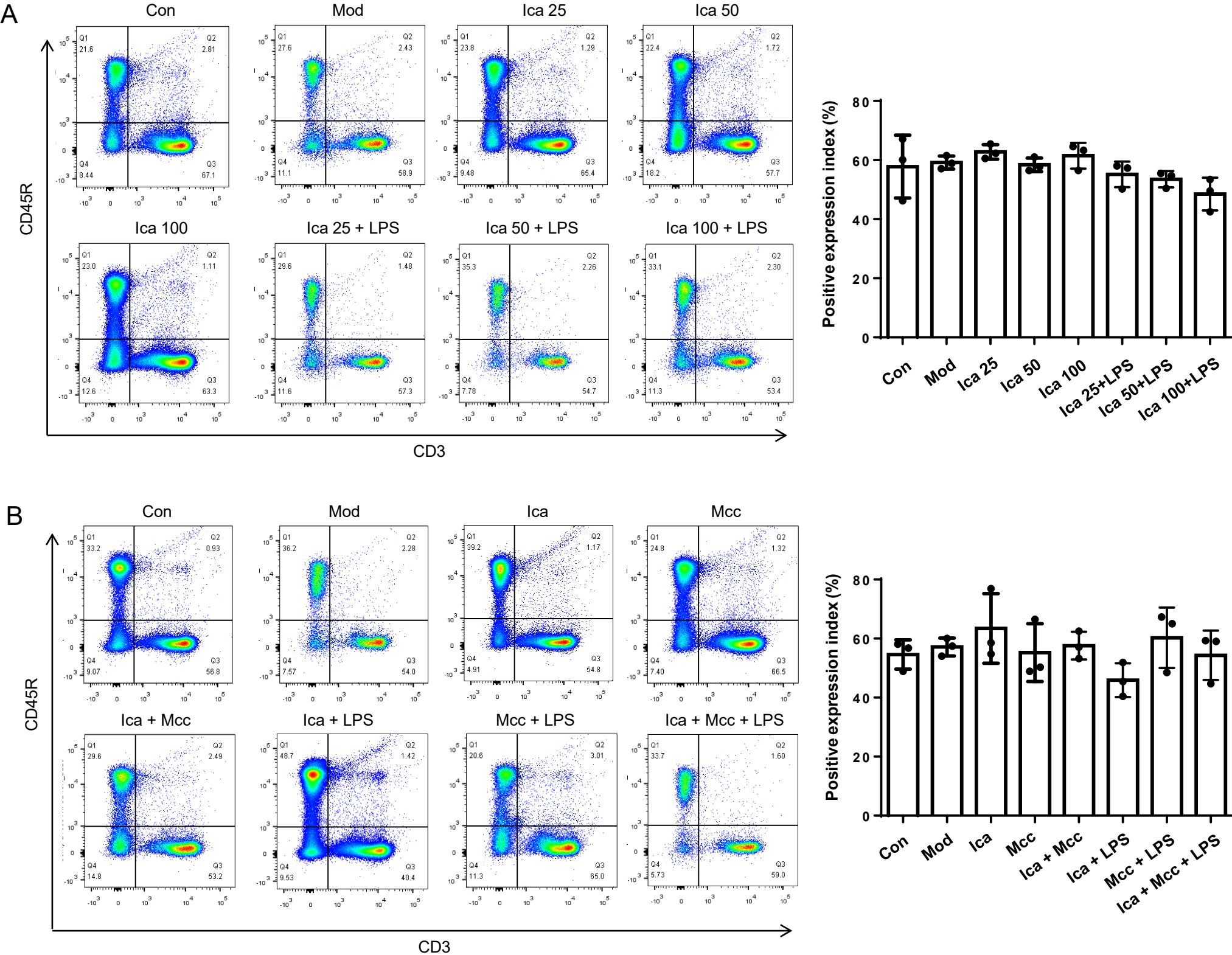

Figure S5

A

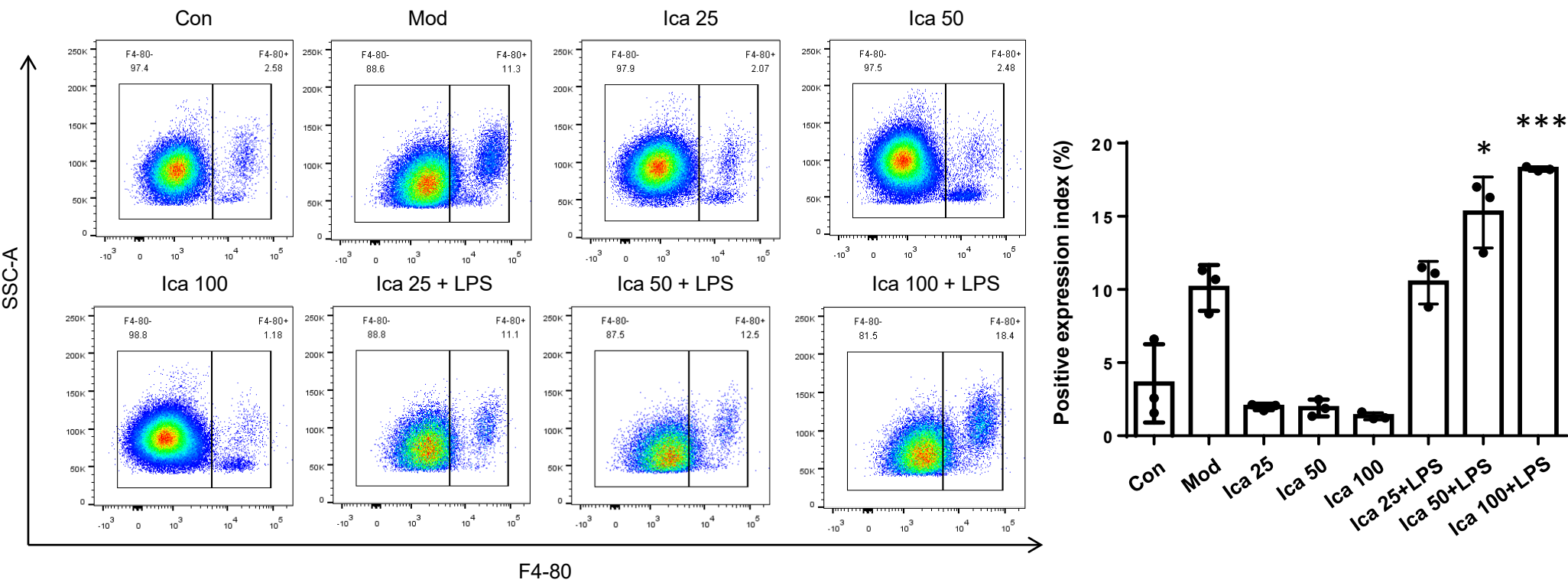

B

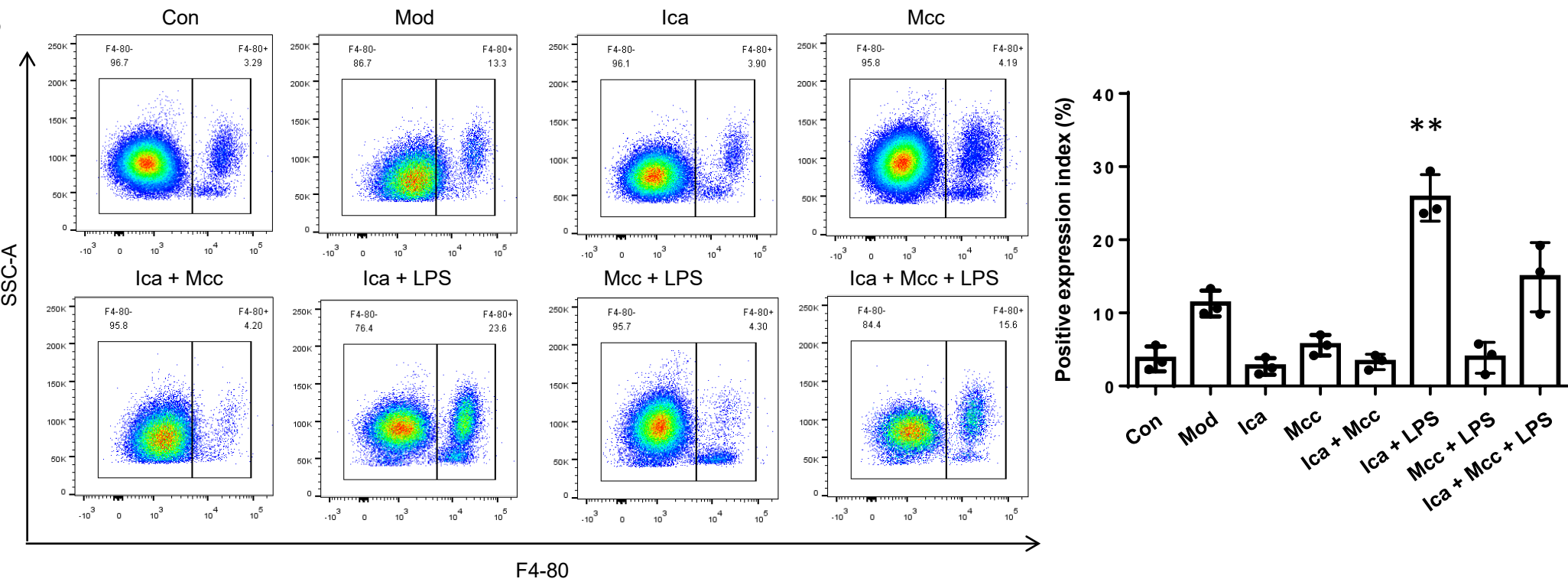

Figure S6

A

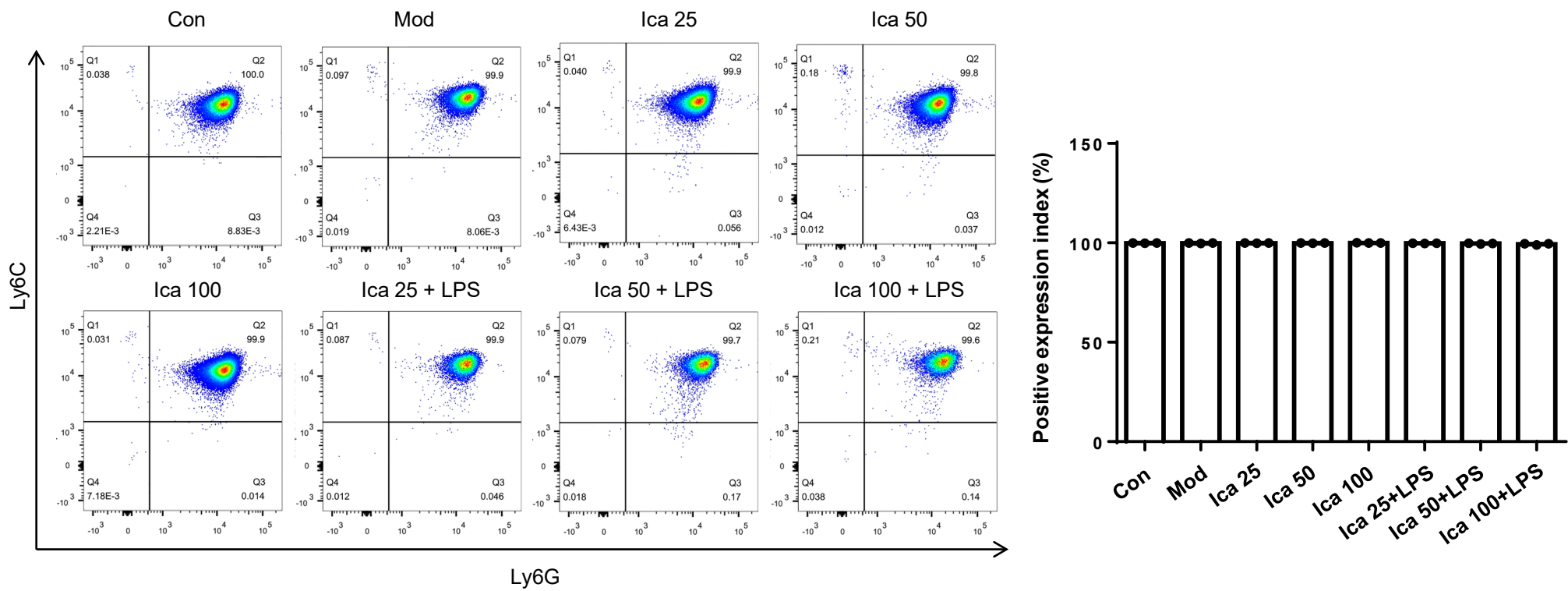

B

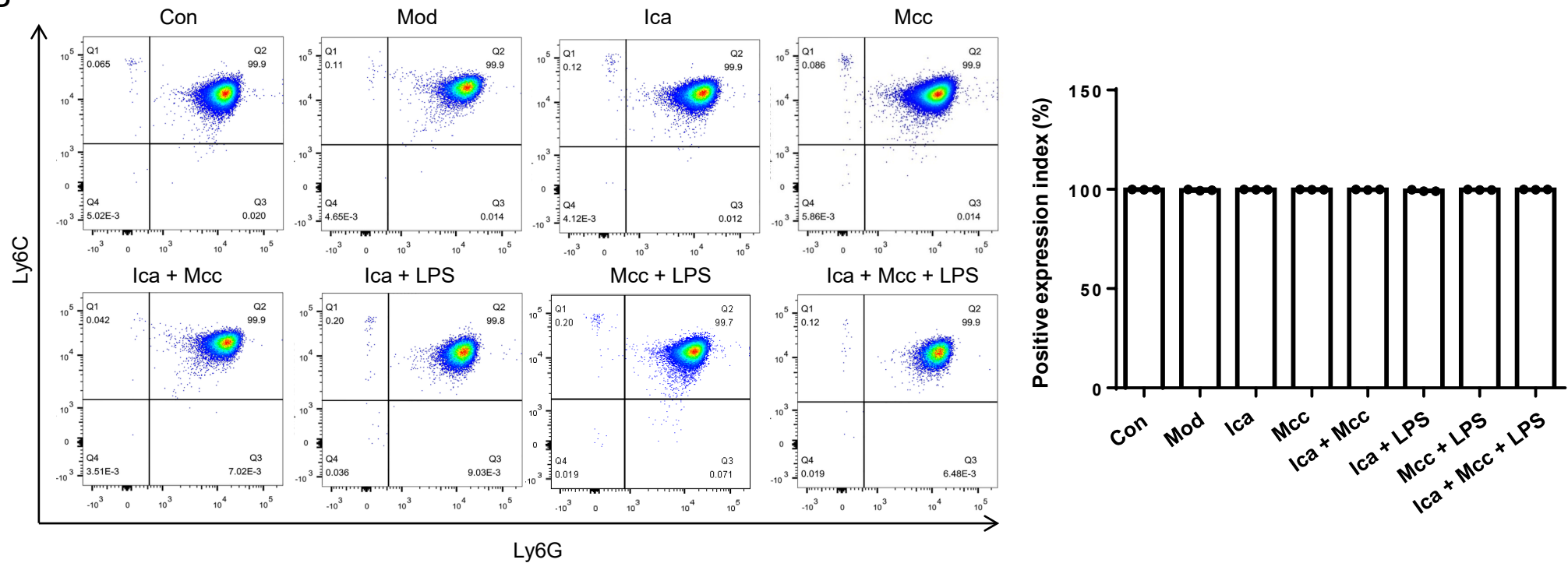

Figure S7

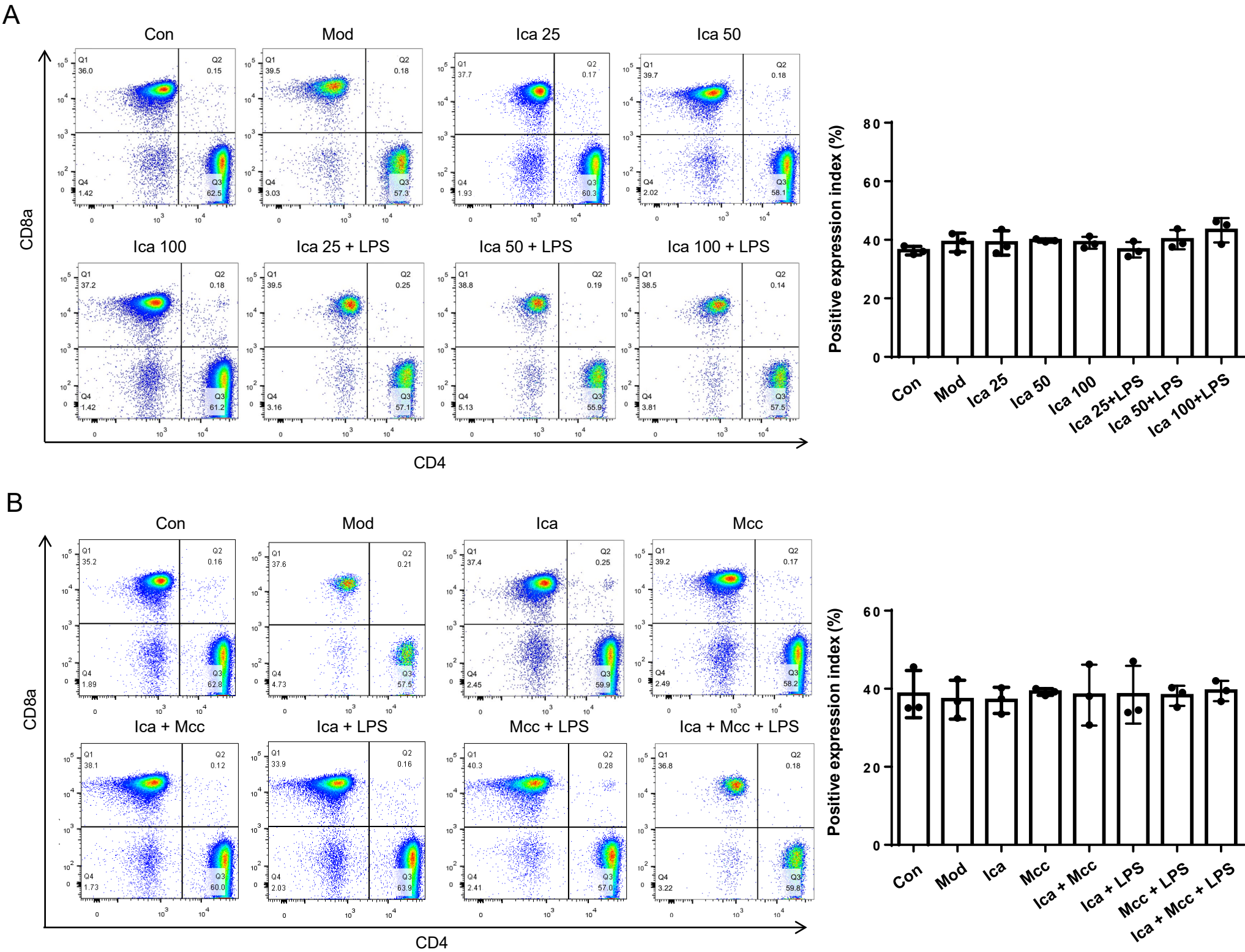

Figure S8

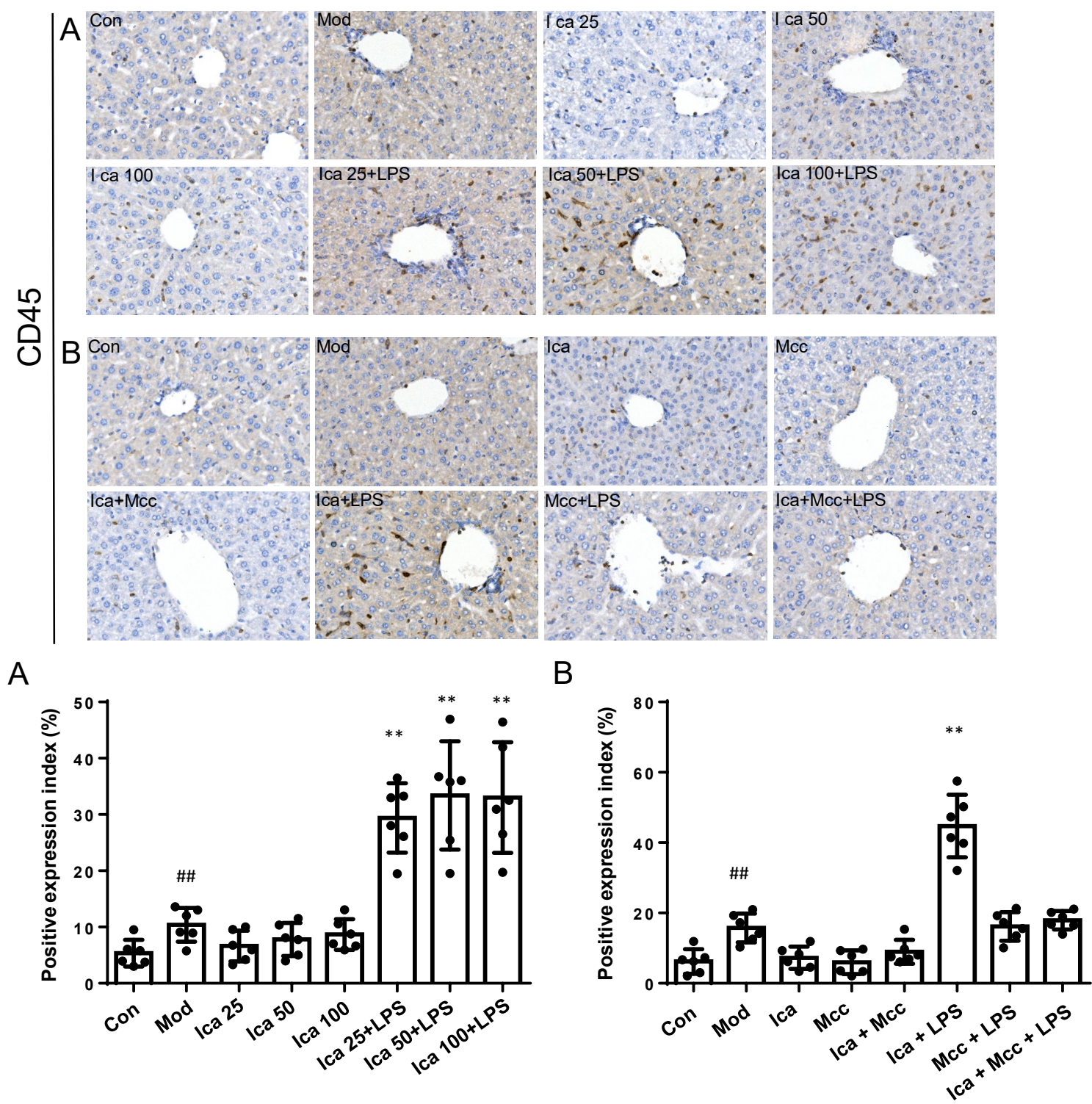

Figure S9

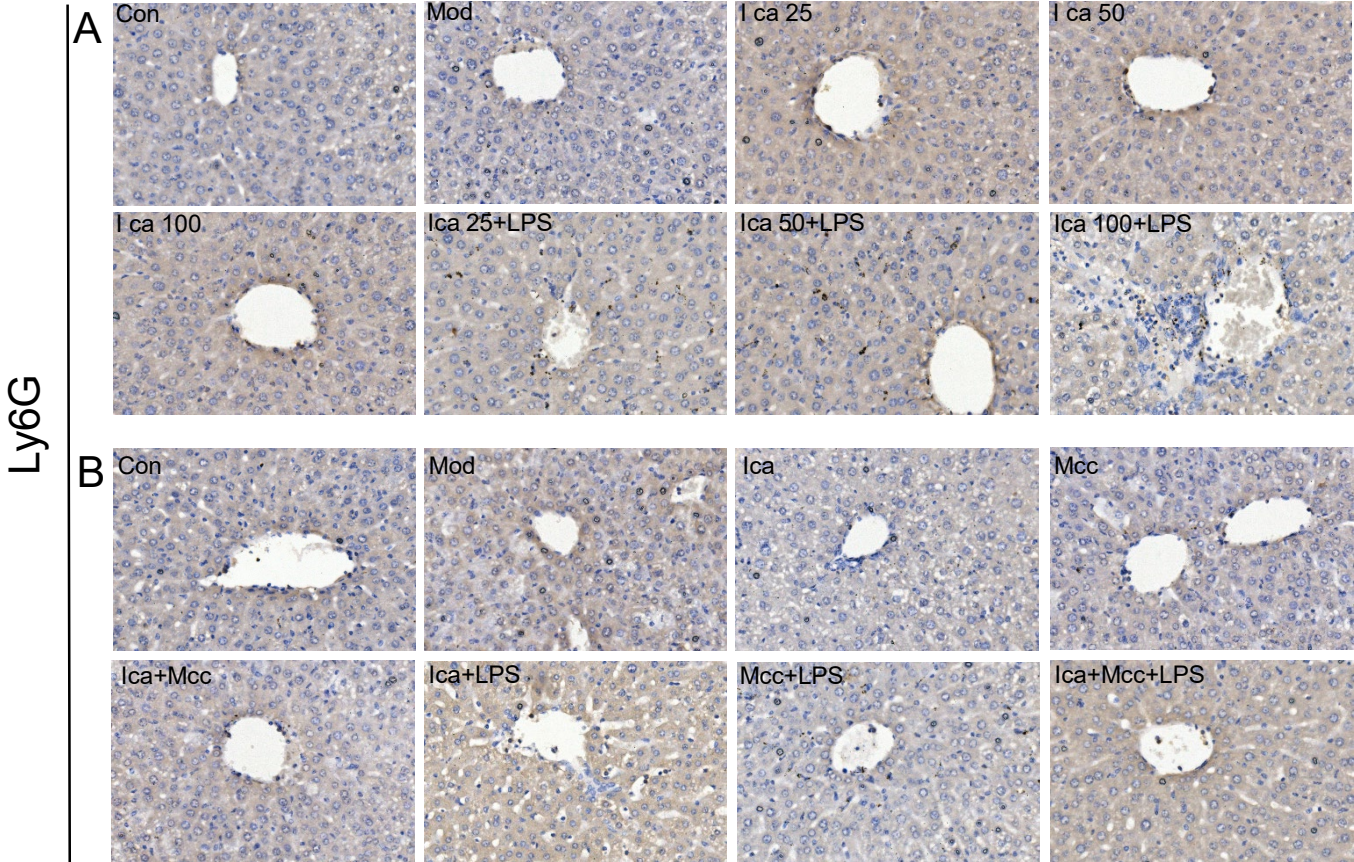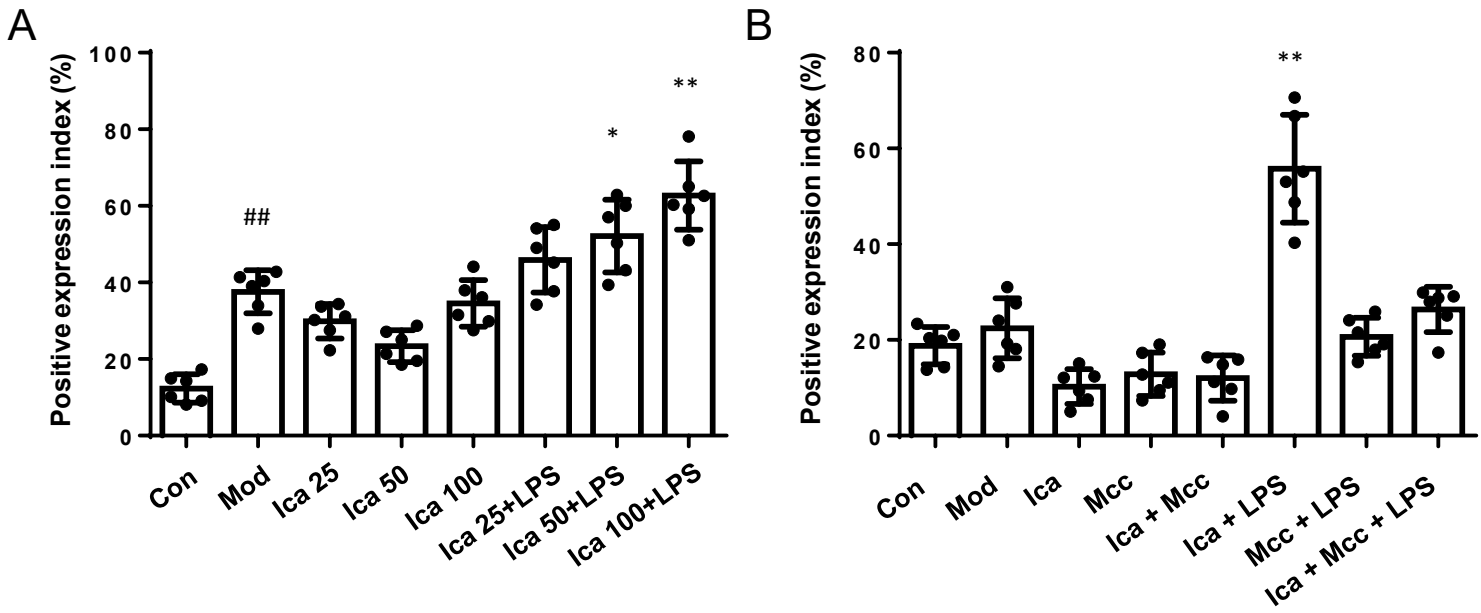

Figure S10

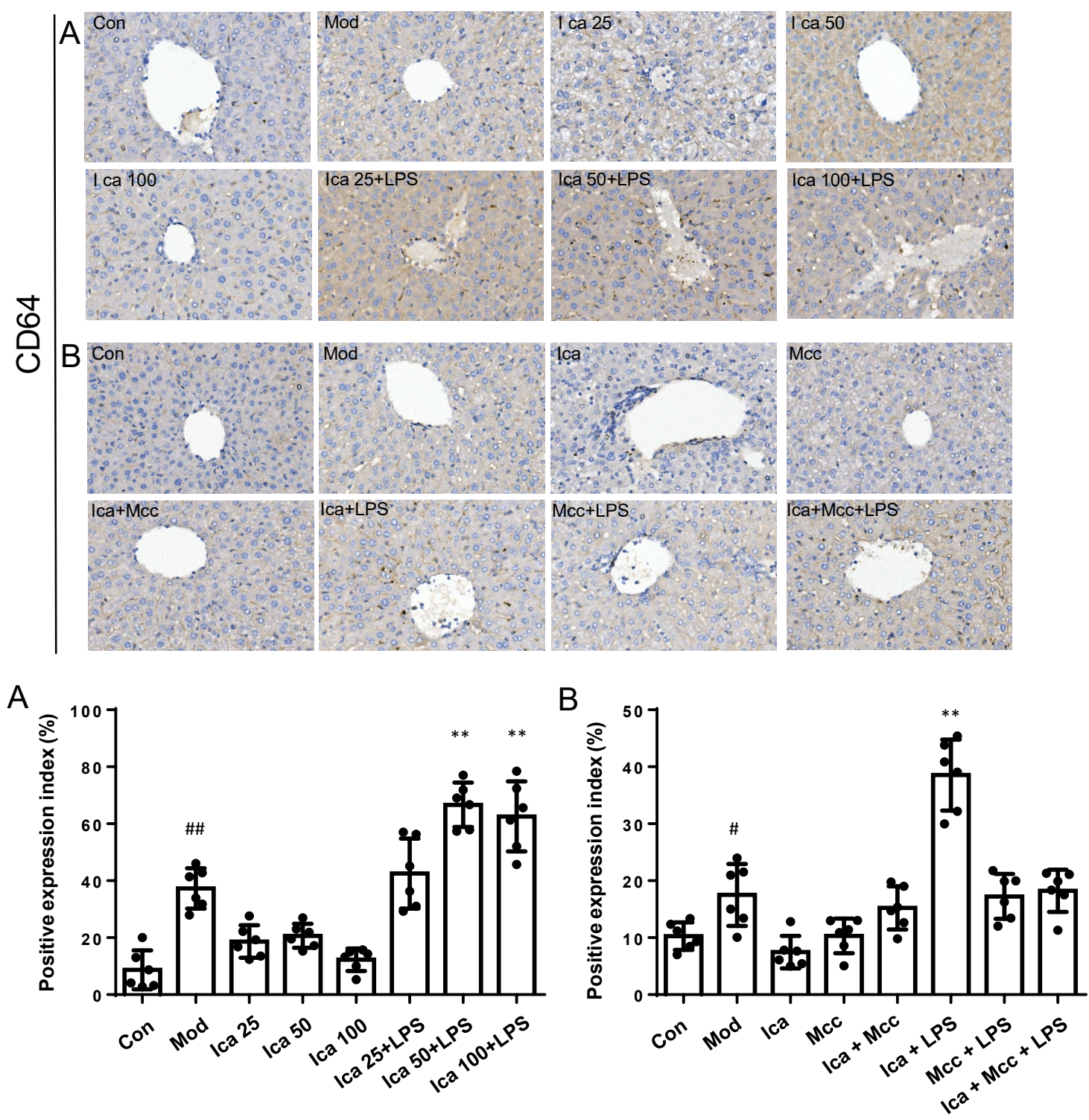

Supplement: Supplementary file 2 — Additional file 1: Figure S1: Icariside I facilitates ATP/nigericin-induced NLRP3 inflammasome activation dependent on mitochondrial ROS production. Flow cytometry was conducted to test mtROS production (two independent experiments). Figure S2: Icariside I doesn’t influence NEK7 interaction with NLRP3 in vitro. Immunoprecipitation (IP) and immunoblot analysis of the interaction of Flag-tagged NLRP3 in the lysates of HEK-293 T cells. Icariside I was added at 6 h post-transfection. Figure S3: Icariside I facilitates ATP/nigericin-induced NLRP3 inflammasome activation by increasing mitochondrial ROS production. (A, B) LPS-primed BMDMs were treated with Icariside I or NAC before stimulated with ATP. BMDMs were loaded with MitoSOX red mitochondrial superoxide indicator (Ex/Em: 510/580 nm). After staining and washing, flow cytometry was conducted to test mtROS production. (C) Western blots of SN and WCL from LPS-primed BMDMs treated with Icariside I, NAC or Icariside I plus NAC before ATP stimulation. (D) Caspase-1 activity in SN from LPS-primed BMDMs treated with Icariside I, NAC or Icariside I plus NAC and then stimulated with ATP. Figure S4: The leucocytes production after Icariside I/LPS cotreatment. Assessed the B cells infiltration by FACS. Figure S5: The leucocytes production after Icariside I/LPS cotreatment. Assessed the macrophages cells infiltration by FACS. Figure S6: The leucocytes production after Icariside I/LPS cotreatment. Assessed the neutrophils and infiltrating monocyte cells infiltration by FACS. Figure S7: The leucocytes production after Icariside I/LPS cotreatment. Assessed the T cells infiltration by FACS. Figure S8: The leucocytes production after Icariside I/LPS cotreatment. Immunohistochemistry experiments in tissue sections (CD45). Figure S9: The leucocytes production after Icariside I/LPS cotreatment. Immunohistochemistry experiments in tissue sections (Ly6G). Figure S10: The leucocytes production after Icariside I/LPS cotreatment. Immunohisto [file 12964_2020_647_MOESM2_ESM.pdf]
